# Low expression of CD39 and CD73 genes in centenarians compared with octogenarians

**DOI:** 10.1186/s12979-017-0094-3

**Published:** 2017-05-19

**Authors:** Almudena Crooke, Juan Martínez-Henández, Joaquín Martínez-López, Alfonso Cruz-Jentoft, Fernando Huete-Toral, Jesús Pintor

**Affiliations:** 1 Department of Biochemistry and Molecular Biology IV, Faculty of Optics and Optometry, Universidad Complutense de Madrid, C/Arcos de Jalón 118, 28037 Madrid, Spain; 2 General Medical Council of Spain, Fundación para la Formación, Madrid, Spain; 3 Department of Hematology, Hospital Universitario 12 de Octubre, Madrid, Spain; 4 Department of Geriatrics, Hospital Universitario Ramón y Cajal, Madrid, Spain

**Keywords:** Ageing, Centenarian, Adenosine receptor, Ecto-nucleotidase

## Abstract

Ageing involves a progressive decline of the body’s regulatory systems including immune system. Adenosine regulates immune function by interaction with its receptors, mainly adenosine A2A receptor, present on the surface of immune cells. Furthermore, cellular response to this nucleoside is highly dependent on its extracellular concentration that is regulated by ecto-enzymes such as CD39 and CD73. Therefore, the aim of this study was to investigate the effect of age on adenosine A2A receptor, CD39 and CD73 gene expression. Changes in mRNA were measured by quantitative PCR from peripheral blood of young, middle-aged and older adults as well as centenarians. Centenarians showed a prominent decrease of CD39 and CD73 mRNA in comparison with older adults. Regarding to adenosine A2A receptor, we detected two subgroups of centenarians with high and low level of transcript. Additionally, adenosine A2A receptor mRNA level of centenarians, did not correlate with their cognitive impairment. In summary, our pilot study suggests that unlike of adenosine A2A receptor, the level of CD39 as well as CD73 mRNA could be a hallmark of successful human ageing.

**Electronic supplementary material:**

The online version of this article (doi:10.1186/s12979-017-0094-3) contains supplementary material, which is available to authorized users.

## Introduction

Immunosenescence, the age-related decline in immune response, increases morbidity and mortality in the elderly population [1]. Therefore, the preservation of immune function is a marker of longevity. Indeed, the function of innate and adaptive immunity cells is not impaired in centenarians, example of extreme longevity and compression of morbidity [2, 3].

Adenosine regulates immune function by interaction with its receptors, mainly adenosine A2A receptor (ADORA2A), present on the surface of immune cells [4].

This nucleoside is generated from extracellular nucleotides by ecto-enzymes such as CD39 and CD73 that are expressed by immune cells [4]. Consequently, these proteins, can also regulate immune function [5].

Whereas these previous studies showed the key role of adenosinergic system in immune regulation, they provided little information about the age-related changes of this system [4, 5]. Therefore, and since immune response is crucial to a healthy ageing, we have investigated the effect of age on CD39, CD73 and ADORA2A mRNA expression in blood cells of young, middle-aged and older adults as well as centenarians.

## Material and methods

### Subjects and samples

A total of fifty-seven healthy subjects from Spain were included in the analysis. We used peripheral blood of 13 young adults (median age: 23, 8 males and 5 females), 12 middle-aged adults (median age: 45, 6 males and 6 females), 11 older adults (median age: 79, 3 males and 8 females) and 21 centenarians (median age: 102, 4 males and 17 females).

Individuals with autoimmune diseases or similar; inflammation; malignancy; malnutrition (Quetelet index < 20 for females and 22 for males); alcoholism and dementia (when it eliminates his consent capability) were excluded. It was evaluated the physical (by modified Barthel index [6]) and mental health (by Spanish version of the Pfeiffer’s test [7]) statuses of older adults as well as centenarians (see Additional file 1).

Peripheral blood samples were collected in BD Vacutainer EDTA tube (BD Biosciences, New Jersey, USA) always in the morning. Blood aliquots were frozen and stored at – 80 °C until total RNA isolation.

### RNA isolation, cDNA synthesis and quantitative PCR

Total RNA was isolated from blood aliquots using NucleoSpin RNA Blood Kit (Macherey-Nagel, Düren, Germany), following the manufacturer’s instructions. First-strand cDNA synthesis was performed from 33 μl of total RNA, using High Capacity cDNA Reverse Transcription Kit and random hexamer primers (Life Technologies, Madrid, Spain). Quantitative PCR (qPCR) was performed in triplicate using cDNA, the Quantitect SYBR Green Kit (Qiagen, Madrid, Spain) and gene-specific PCR primers for CD39 (QT00094787, Qiagen), CD73 (QT02451512, Qiagen) as well as ADORA2A (5′-GGATGTGGTCCCCATGAACTA-3′/5′-CCAGGAAGATCCGCAAATAGAC-3′), on an ABI Prism 7300 PCR System (Life Technologies). The thermal cycler program was 15 min at 95 °C, followed by 40 cycles of 15 s at 94 °C, 30 s at 55 °C and 34 s at 72 °C (data collection step). Non-template and non-reverse transcribed controls were included in all experiments. Analysis of the melting curves confirmed the specificity of PCR and the absence of primer-dimers.

β-actin gene (ACTB) (HK-SY-hu-600, PrimerDesign, Southampton, UK) was used as internal control to normalize mRNA relative expression, after its validation for qPCR. Validation of the internal control gene and qPCR data analysis were performed by 2^-ΔCt^ and 2^-Δ∆Ct^ method, respectively, once confirmed that the amplification efficiency of all primers pairs were similar and close to a value of 2 (Additional file 1).

### Statistical analysis

GraphPad Prism 6.0 (GraphPad Software, San Diego, USA) was used for statistical analyses, and the tests used are given in the corresponding figure legends. The adequacy of sample size was tested using the statistical software Granmo 6.0 (Institut Municipal d’Investigació Médica, Barcelona, Spain) with an accepted two-sided alpha risk of 0.05 and a beta risk of 0.20.

More information in Additional file 1.

## Results

We examined the mRNA level of CD39, CD73 and ADORA2A by qPCR, using ACTB gene to normalize because its expression was unaffected by age (*p* = 0.7627) (Additional file 2: Figure S1).

**Fig. 1:**
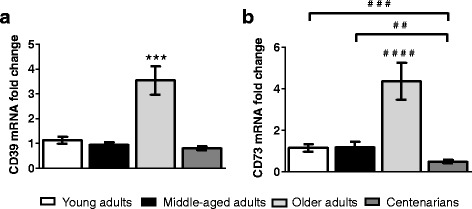
Effect of ageing on CD39 and CD73 gene expression in human peripheral blood cells. Quantification of CD39 (**a**) and CD73 (**b**) mRNA fold change in cells of young, middle-aged and older adults as well as centenarians. Gene expression level data for each gene and sample were normalized to ACTB signal (internal control) and relative to the mean normalized value of CD39/CD73 gene expression in young adults group. Error bars represent S.E.M. of the means obtained in each group (*n* = 13 per young adults and centenarians; *n* = 12 per middle-aged adults and *n* = 11 per older adults group). **a**: ^***^ indicates statistically significant differences (*p* < 0.001) between older adults and the rest of groups. **b**: ^****^ denotes highly significant differences (*p* < 0.0001) between older adults and the rest of groups; ^# #^ and ^# # #^ mean significant differences (*p* < 0.01 and *p* < 0.001, respectively) between groups linked by horizontal square brackets. The *p* values were determined by Mann–Whitney test

**Fig. 2:**
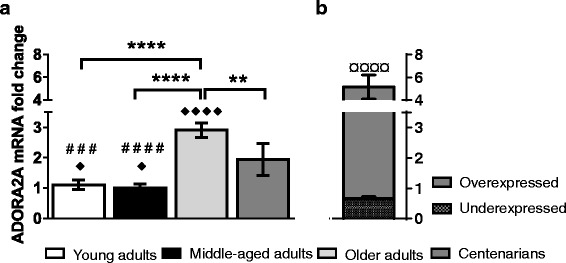
Effect of ageing on ADORA2A gene expression in human peripheral blood cells. **a** Quantification of ADORA2A mRNA fold change in cells of young, middle-aged and older adults as well as centenarians. ADORA2A mRNA level for each sample was normalized to ACTB signal (internal control) and relative to the mean normalized value of ADORA2A gene expression in young adults group. Error bars represent S.E.M. of the means obtained in each group (*n* = 13 per young adults; *n* = 12 per middle-aged adults; *n* = 11 per older adults; *n* = 21 per centenarian group). ** (*p* < 0.01) and **** (*p* < 0.0001) indicate statistically significant differences between older adults and the rest of groups. **b** ADORA2A mRNA fold change in different subgroups of **a** centenarians. ^¤ ¤ ¤ ¤^ (*p* < 0.0001) indicates significant differences between centenarian’s subgroups, overexpressed (*n* = 7) and underexpressed (*n* = 14). ^# # #^ (*p* < 0.001) and ^# # # #^ (*p* < 0.0001) denote highly significant differences between the subgroup of overexpressed centenarians and young as well as middle-aged adults groups of **a**, respectively. ^♦^ (*p* < 0.05) and ^♦ ♦ ♦ ♦^ (*p* < 0.0001) mean statistical differences between the subgroup of underexpressed centenarians and the rest of groups of **a**. The *p* values were determined by Mann–Whitney test

In our study, young and middle-aged individuals presented a similar pattern of expression for all genes (*p* > 0.05) (Figs. 1 and 2a).

We have found that centenarians have a comparable level of CD39 mRNA (*p* > 0.05) and a lower level of CD73 mRNA (2.3-fold lower) than young adults (Fig. 1). Conversely, older adults showed a marked overexpression of CD39 (3.1-fold vs. young and 4.4-fold vs. centenarians) and CD73 (3.8-fold vs. young and 8.8-fold vs. centenarians) genes (Fig. 1). Likewise, we observed ADORA2A gene overexpression in older adults vs. young adults (2.6-fold) and centenarians (1.5-fold) (Fig. 2a).

Due to the abnormal error bar of centenarians group (Fig. 2a), we analyzed the level of ADORA2A mRNA in each centenarian sample and detected two subpopulations: one with a high level of transcript (so-called “overexpressed” centenarians) and other with a low level of transcript (so-called “underexpressed” centenarians) (Fig. 2b). Overexpressed centenarians presented higher ADORA2A mRNA level than young (4.6-fold) and similar level to older adults (*p* = 0.5804) (Fig. 2). Conversely, underexpressed centenarians showed a low level of ADORA2A transcript in comparison with young (1.5-fold lower) and older adults (4.4-fold lower) (Fig. 2). Nevertheless, the difference with young group is statistically inconclusive due to the sample size.

Finally, we also studied individual characteristics including presence or type of chronic disease, commonly used drugs, gender, the level of physical function and cognitive impairment, in the two subgroups of centenarians. We found only differences, but not statistically significant, in the number of individuals with mild and moderate impairment (*p* = 0.073) (Table 1).

**Table 1:** Proportion of cognitive impairment in centenarian subgroups

Mental status	Overexpressed, % (n)	Underexpressed, % (n)
Intact intellectual functioning (0–2 errors)	14.3 (1)	38.5 (5)
Mild intellectual impairment (3–4 errors)	0.0 (0)	23.1 (3)
Moderate intellectual impairment (5–7 errors)	57.1 (4)	7.7 (1)
Severe intellectual impairment (8–10 errors)	28.6 (2)	30.8 (4)

Mental status was determined by the Spanish version of the Pfeiffer’s test [7]. *p* = 0.073, Chi-square test

## Discussion

Ageing involves a progressive decline of the immune system [8]. Since adenosine is essential for immune function [4] we have investigated the effect of age on CD39 and CD73, as well as ADORA2A mRNA expression in human blood cells [9, 10].

Our results show lower expression of CD39 and CD73 genes in centenarians than older adults. In Spain, only 4% of people aged 70 will be centenarians (data obtained from 1996 and 2016 census of the National Statistics Institute, Spanish Statistical Office). Since centenarians are an example of compression of morbidity and extreme longevity, underexpression of these genes might be an important factor to successful ageing [11]. Indeed, centenarians present immune cells with a similar activity to those of young people [2, 3, 12]. These immunological circumstances might explain, at least in part, why centenarians can keep out infections and thus prolong their life.

Age-associated changes of immune cells at the molecular level have also been studied. So, Serna and colleagues demonstrated a similar pattern of mononuclear cells small non-coding RNAs expression between centenarians and young people whereas that of octogenarians was radically different [13]. Additionally, Fann and coworkers analyzed the gene-expression profiles of human memory CD8^+^ T cells and found changes in the expression of several cell-surface receptors such as CD28 with age [14]. More recently, Fang and colleagues have found CD39 overexpression in stimulated T cells of older individuals [15]. These authors demonstrate that the age-mediated induction of CD39 mRNA and its cognate protein evoke T cell apoptosis and thus, prevents the generation of memory T cells after vaccination [15]. Indeed, CD39 cleaves ATP to ADP and AMP that via P2 purinergic receptors may control cellular immune response. CD73 degrades AMP to adenosine that also mediate purinergic effects via its P1 receptors [10]. Mackiewicz and colleagues observed a higher CD73 activity in the brain of older rats than in young animals [16]. Additionally, several studies have demonstrated that CD73 is overexpressed in cancer, a disease that predominantly affects older individuals [17, 18].

Our qPCR results also demonstrate CD73 underexpression in centenarians versus young adults. Previous studies have found a decline of centenarians CD28^+^CD8^+^ T cells when compared with those of young people [19, 20]. CD73 is expressed on the surface of CD8^+^ T lymphocytes and therefore, the smaller number of these cells in centenarians might explain why they underexpress CD73 [21, 22]. Additionally and since centenarians present a greater number of CD28^+^CD8^+^ T cells than octogenarians, the differences of expression between these last groups could be larger than those observed [19].

Regarding ADORA2A, our results show that older adults present a higher level of ADORA2A transcript than young adults and centenarians. Again, high expression of this gene seems to be a handicap to a healthy ageing. In fact, overexpression of ADORA2A mediate the susceptibility to apoptosis of older CD39^+^ T cells [15].

Nonetheless, differences between older adults and centenarians are lesser than those for CD39/CD73. Indeed, centenarians present higher but not significant ADORA2A expression than young adults. These results can be explained by the existence of two subpopulations of centenarians characterized by high and low level of ADORA2A mRNA. Consequently, the level of ADORA2A mRNA could not be a critical factor for successful ageing.

Evidence of the existence of different centenarian subpopulations has been previously reported [23]. This work defined two subgroups of centenarians with high and low values of basic immune parameters [23]. On the other hand, the group of Bergamaschini detected different levels of ADORA2A mRNA and its cognate receptor in patients with diverse grade of cognitive impairment [24]. Our statistical analysis on mental damage in both centenarian subgroups indicates, that their grade of cognitive impairment, is independent of ADORA2A mRNA levels.

It must be stated that this is a preliminary study and more research is needed to confirm our results. We propose to investigate the relationship between mRNA and protein expression, particularly in the case of ADORA2A. Additionally, an excellent subject of study is the possible correlation of our gene expression findings with the number and type of immune cells. The study of centenarian offspring is currently a focus of attention for much longevity research [25]. In fact, Van den Berg and colleagues have recently demonstrated that individuals with a familial predisposition for longevity present a high-amplitude rhythm of serum cholesterol concentration [26]. In this context, numerous studies have provided the connection between circadian rhythms and the immune system [27, 28]. Therefore, we think that it would be very interesting to carry out this same study in centenarian’s offspring.

## Conclusions

Our analysis of peripheral blood gene expression demonstrated a significantly lower level of CD39 and CD73 mRNA in centenarians than older adults. Consequently, we suggest that the level of CD39 as well as CD73 mRNA could be a hallmark of successful human ageing.

## Additional files

Additional file 1: Material and Methods. (DOCX 13 kb)

Additional file 2: Figure S1. Validation of ACTB gene as internal control for quantitative PCR assays. Age-related changes on ACTB gene expression in human peripheral blood cells. ACTB gene expression in young adults is set to 1 (calibrator sample). Each data point represents mean of fold change ± S.E.M. (*n* = 13 per young, middle-aged and older adults; *n* = 21 per centenarian group). *p =* 0.7627, One-way ANOVA test. (PPTX 45 kb)
