# Supplementary material for: Low expression of CD39 and CD73 genes in centenarians compared with octogenarians
Source: Immun Ageing. 2017 May 19;14:11. doi: 10.1186/s12979-017-0094-3 (PMC5437401; doi:10.1186/s12979-017-0094-3)
Supplement: Supplementary file 1 — Material and Methods. (DOCX 13 kb) [file 12979_2017_94_MOESM1_ESM.docx]

**Additional file 1.** Material and Methods:

**Subjects**

Subjects included in the analysis were from the central part of Spain (*Comunidad de* *Madrid, Castilla-León and Castilla La Mancha*).

We used two groups of healthy blood donors from the Hospital Universitario 12 de Octubre of Madrid: one of 13 young adults (age range: 20-37) and one of 12 middle-aged adults (age range: 40-56). In addition, a group of 11 healthy older adults (age range: 70-87), volunteers from the Hospital Universitario Ramón y Cajal of Madrid were enrolled. Finally, the last group was formed by 21 centenarians (age range: 100-106) who participated in the Spanish Centenarian Study Group “RENACE” sponsored by the “Sociedad Española de Médicos Generales y de Familia”.

38% of centenarians and 100% of older adults are moderate or totally independent (Barthel index score > 60). 48% of centenarians and 91% of older adults have normal intellectual functioning or mild cognitive impairment (Pfeiffer´s test score < 5).

**RNA isolation, cDNA synthesis and quantitative PCR**

Amplification efficiency of ACTB, CD39, CD73 and ADORA2A primers pairs were: *E_ACTB_= 2.03, E_CD39_=* 2.08, *E_CD73_= 2.06* and *E_ADORA2A_*= 2.03. *r^2^≥* 0.997.

**Statistical analysis**

Kolmogorov-Smirnov and ANOVA test were used for validation of the internal control gene. Kolmogorov-Smirnov and Mann-Whitney tests were used for CD39, CD73 and ADORA2A expression analysis. Finally, chi-square test was performed to evaluate the possible association between the level of ADORA2A mRNA and grade of cognitive impairment.
