# Supplementary figures and images for: Low expression of CD39 and CD73 genes in centenarians compared with octogenarians
Source: Immun Ageing. 2017 May 19;14:11. doi: 10.1186/s12979-017-0094-3 (PMC5437401; doi:10.1186/s12979-017-0094-3)

## Slide 1
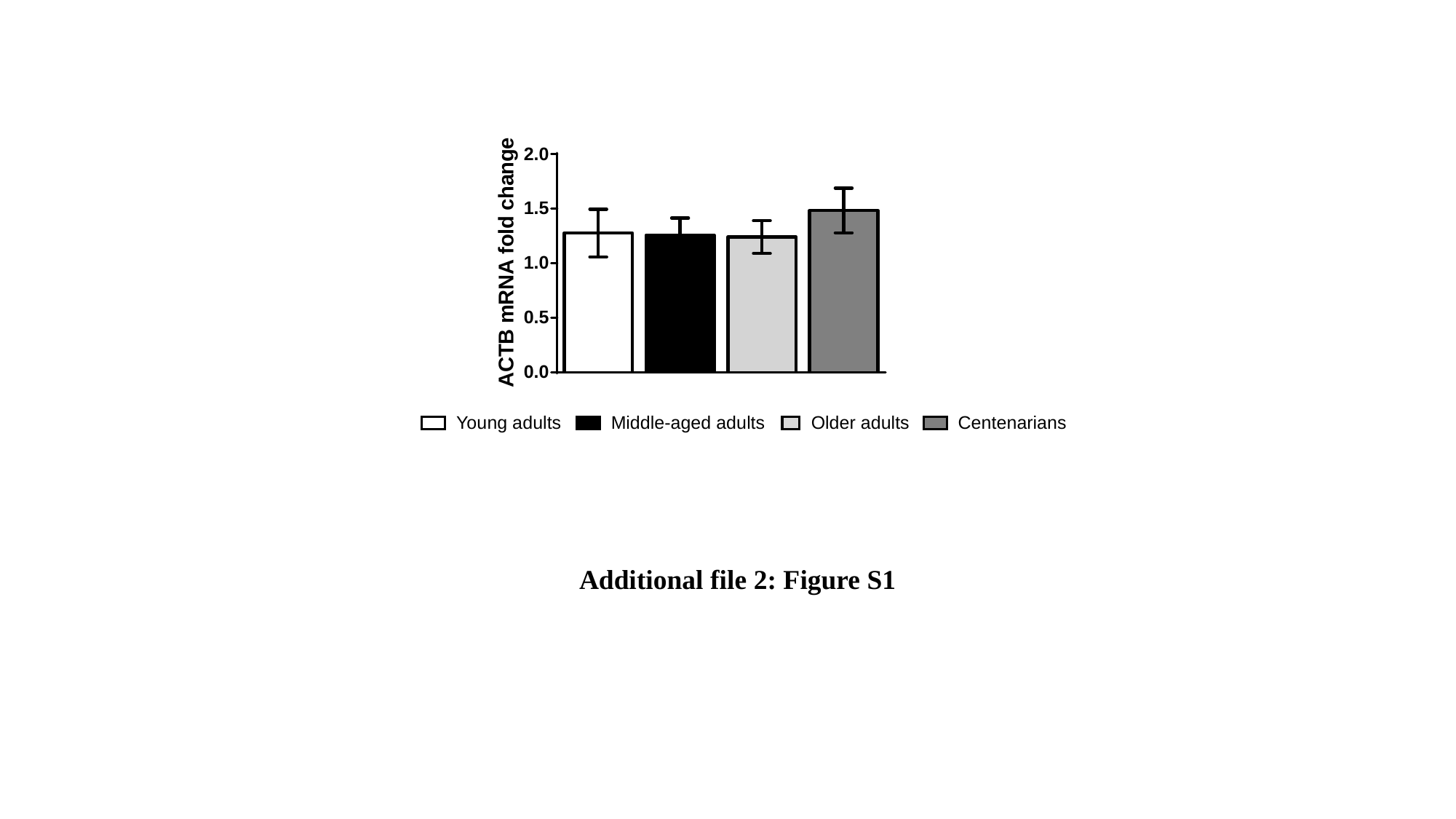

Young adults
Middle-aged adults
Older adults
Centenarians
Additional file 2: Figure S1

Supplement: Supplementary file 2 — Validation of ACTB gene as internal control for quantitative PCR assays. Age-related changes on ACTB gene expression in human peripheral blood cells. ACTB gene expression in young adults is set to 1 (calibrator sample). Each data point represents mean of fold change ± S.E.M. (n = 13 per young, middle-aged and older adults; n = 21 per centenarian group). p = 0.7627, One-way ANOVA test. (PPTX 45 kb) [file 12979_2017_94_MOESM2_ESM.pptx]
